# Collaborative study of thresholds for mutagens: proposal of a typical protocol for detection of hormetic responses in cytotoxicity tests

**DOI:** 10.1186/s41021-018-0108-1

**Published:** 2018-10-08

**Authors:** Shizuyo Sutou, Akiko Koeda, Kana Komatsu, Toshiyuki Shiragiku, Hiroshi Seki, Kohji Yamakage, Takeru Niitsuma, Toshiyuki Kudo, Akihiro Wakata, (The Mammalian Mutagenicity Study Group,

**Affiliations:** 1 School of Pharmacy, Shujitsu University, 1-6-1 Nishigawara, Naka-ku, Okayama-shi, 703-8516 Japan; 2 Ina Research Inc., 2148-188 Nishiminowa, Ina-shi, Nagano-ken, 399-4501 Japan; 3 Tokushima Research Institute, Otsuka Pharmaceutical Co., Ltd., 463-10 Kagasuno, Kawauchi-cho, Tokushima, Tokushima-ken 771-0192 Japan; 4 Safety Studies Section, BML Inc., 1361-1 Matoba, Kawagoe-shi, Saitama-ken, 350-1101 Japan; 5 Hatano Research Institute, Food and Drug Safety Center, 729-5 Ochiai, Hadano-shi, Kanagawa-ken 257-8523 Japan; 6 Tsukuba Research Center, Astellas Pharma Inc., 21 Miyukigaoka, Tsukuba-shi, Ibaraki-ken 305-0841 Japan

**Keywords:** Adaptive response, CHL/IU, Ethyl methanesulfonate, HeLa S3, Hormesis, Mitomycin C, TK6

## Abstract

**Background:**

According to the linear no-threshold model (LNT), even the smallest amount of radiation is hazardous. Although the LNT is not based on solid data, this hypothesis has been applied to mutagens and carcinogens. As a result, it has been postulated that there are no thresholds for these chemicals. To demonstrate negativity by experiments is practically impossible, because negative data may leave behind the possibility that additional data might make the resolution power high enough to change negativity to positivity. Furthermore, additional data collection may be endless and we may be trapped in agnosticism. When hormesis is established, in which biological responses are higher at low-doses and lower at high-doses than the control, thresholds could be established between the low- and high-doses. Before examination of thresholds in chemical mutagenesis, hormetic responses in cytotoxicity were tested using cultured mammalian cells.

**Method:**

Human cells (HeLa S3 and TK6) or Chinese hamster cells (CHL/IU) were cultured in 96-well plates and treated with mitomycin C (MMC) or ethyl methanesulfonate (EMS) at various dose levels and optical density was measured after addition of a reagent to detect cellular activity. In hormetic responses, data might fluctuate to and fro; therefore, experimental conditions were examined from various aspects to eliminate confounding factors including cell numbers, detection time, the edge effect of 96-well plates, and measurement time after addition of the reagent for detection.

**Results:**

The dose response relationship was never linear. Cellular activities after treatment with MMC or EMS were generally higher at lower doses levels and lower at higher doses than the control, showing hormesis and allowing the establishment of thresholds. Dose response curves sometimes showed two or three peaks, probably reflecting different cellular responses.

**Conclusion:**

Hormetic responses in cytotoxicity tests were observed and thresholds could be established. Based on the results of this investigation, we put forward a tentative protocol to detect chemical hormesis in cytotoxicity tests, i.e., inoculate 2000 cells per well, add various doses of a test chemical 48 h after inoculation, add a detection dye 10 h after treatment, and measure optical density 2 h after addition of the reagent for detection.

## Background

The linear no-threshold model (LNT) is a hypothesis that postulates that even the smallest amount of radiation is hazardous and is proportional to dose levels. The origin of this hypothesis dates back to 1927 when Muller found that X-rays induced sex-linked recessive lethality in *Drosophila melanogaster* [1]. Apparent linearity at higher doses was extrapolated to lower doses without experimental data. Then in 1939, World War II (WWII) broke out and the United States of America (USA) began production of the A-bomb under its Manhattan Project, and the effects of radiation on living organisms were investigated intensively. *D. melanogaster* irradiated with low-dose radiation showed that there is a threshold in recessive lethality [2]. The USA dropped A-bombs on Hiroshima and Nagasaki in 1945, and Muller became a Nobel laureate in 1946 for his radiation research. Although he must have known there were thresholds, he declared in his Nobel Prize lecture that there was “no escape from the conclusion that there is no threshold dose” [3].

Standard Oil Co. Inc. was founded by John Rockefeller in 1870, who also established the Rockefeller Foundation (RF) in 1913. The oil-industry might well have felt threatened by the discovery of atomic energy. The Republican Party had forged a close relationship with the oil industry, but it was the Democratic Party, led by F.D. Roosevelt (1933–1945) and H. Truman (1945–1953), that governed USA during and after WWII. When the Republicans were reelected, Nelson Rockefeller was appointed as an important aid to President D.D. Eisenhower. Muller, in turn, had close ties to the RF, and in 1954, the RF chose to finance a big project to evaluate ionizing radiation [4].

RF asked the U.S. National Academy of Sciences (NAS) to organize the whole program, which was conducted under the auspices of Bronk, president of NAS and Rockefeller University, and an RF trustee. The Genetics Panel (GP) of the NAS Biological Effects of Atomic Radiation (BEAR) committee was established in 1954 and was chaired by Weaver, a mathematician and fellow RF officer.

Without significant discussion, GP recommended LNT on June 12, 1956 [5]. This document was anonymous. The limit dose for nuclear workers of 500 mGy/y, that had been in place since 1934, was discarded. The next day, the New York Times, owned by a RF trustee, reported that radiation is dangerous on the front page. Other media followed. Soon, several leading biologists asked GP to provide documentation that supported LNT. GP refused to do so, because they never possessed any relevant data. This was accused, reasonably-so, to be an ideologically motivated decision based on deliberate falsification and fabrication of research records [6].

Lewis (later, a Nobel laureate) argued in 1957 that radiation-induced leukemia followed the LNT hypothesis [7]. There are several prominent researchers who indicated flaws in Lewis’ paper (Table 2 in ref. [8]). In fact, leukemia in A-bomb survivors shows hormesis [9]. Thus, data obtained using the sperm of fruit flies, which do not possess repair mechanisms, were applied to human somatic cells that are proficient in repair, apoptosis, immune functions, and other defensive mechanisms. Someone said that the prediction of human cancer using insect sperm data almost comes under the category of ‘man bites dog.’

Another crucially important document is the so-called BEIR VII report published by the NAS Biological Effects of Ionizing Radiation (BEIR) committee [10]. It uses the life span study (LSS) of A-bomb survivors, which is considered to be the most important data source to estimate radiation effects on humans, as the principal data supporting LNT. This report employs the same trick as Lewis [7] to hide evidence of a threshold by combining lower exposure zones. The BEIR VII report cherry-picks the first part of a reference that DNA double-strand breaks induced by very low dose radiation cannot be repaired and quotes that this supports LNT. It omits, however, the succeeding statement of this reference, that breaks induced by high dose radiation are efficiently repaired, which refutes LNT [11]. The report also erroneously draws linearity from experimental data that shows both hormesis and thresholds [12]. The Bayesian analyses of LSS [13] show that the dose-response relationship is sigmoidal, a threshold can be established, and hormetic responses are seen at lower doses, thus refuting LNT [14]. The latest analyses [15] of LSS severely criticize that LNT is no longer tenable [16, 17]. Exposure doses in LSS have been largely underestimated and the incidence of cancer was overestimated accordingly [18]. However, the two key documents supporting LNT (LNT recommendation [5] and BEIR VII report [10]) were presented by NAS, the highest authority in the scientific world and therefore LNT is still dominating radiation regulations to date.

After LNT was proposed to be applicable to cancer induction by ionizing radiation, chemical carcinogenesis attracted interest and the somatic mutation hypothesis (SMH) became the central concept in carcinogenesis. Ames and his colleagues developed the *Salmonella* bacterial test system for screening mutagens. In the 1970s, the notion “carcinogens are mutations” prevailed and supported the SMH [19]. The U.S. Food and Drug Administration (FDA) and Environmental Protection Agency (EPA) played a pivotal role in applying LNT to chemical carcinogens.

Even though LNT was not supported by valid data [20] and radiation hormesis and thresholds were established in *Drosophila* irradiated with X-rays [21] or γ-rays [22], the LNT hypothesis has been maintained for decades not for scientific but for political reasons. The failed LNT is still applied to mutagens/carcinogens today. As for thresholds in chemical treatments, let us introduce several relevant papers.

The *Salmonella typhimurium* tester strain, TA1535, was modified to obtain three DNA repair-deficient strains and mutagenicity testing was carried out [23]. Mutagens induced much more mutants in these three strains than the parental strain of TA1535, indicating that mutations are suppressed by repair mechanisms in bacteria. LNT does not take into account the fact that DNA lesions can be repaired.

Similarly, thresholds in bacterial mutations were studied by Watanabe [24] by applying experimental results obtained using repair-proficient and -deficient strains to formulae which integrated detoxification and repair variables. The conclusion is that risk assessment using the LNT model is possible and the threshold concept should not be used. This study contains some shortcomings: 1) mutations are not controlled by only two factors (detoxification and repair); 2) only two test chemicals were used (N-methyl-N′-nitro-N-nitrosoguanidine (MNNG) and sodium azide), meaning that DNA lesions were limited; 3) repair-deficient genes are confined to three genes and far from sufficient for the evaluation of thresholds; 4) genomic structure is different in prokacryotes and eukaryotes, e.g., introns/exons and chromatin; and 5) mammals have more sophisticated defense mechanisms, such as apoptosis and immune systems, that would nullify mutagenic effects. It is of interest to point out that MNNG induced J-shaped curve responses in TA1535 (Fig. 3b in [24]), indicating that bacteria also seem to show hormesis.

When mutations in the introns and exons of murine *p53* gene were examined, greater mutability of introns over exons was detected, suggesting some selective mechanisms were at work [25]. The authors also showed that repair-defective cell lines from xeroderma pigmentosum were more sensitive to aneugens than normal human fibroblast cells. Normal human cells must have some system to reduce the hazardous effects of xenobiotic chemicals.

Mutagenicity testing — typically, the Ames test, chromosomal aberration or gene mutation test in cultured mammalian cells, and the micronucleus test in rodents — is obligatory in the development of drugs. When testing gives positive results, further development of that candidate drug is often terminated. This is a great loss for human health. Researchers at pharmaceutical companies scrutinized genotoxicity testing, with special attention to the differences between in vitro and in vivo testing [26]. Taking into consideration the wide variety of possible mechanisms affecting in vivo thresholds, the authors conclude that genotoxic damage found in vitro would not be reflected in vivo, and that thresholds could be established in vivo. Supporting evidence for thresholds is also presented.

In spite of these supporting data, risk assessments of genotoxic chemicals are presently based on LNT, i.e., the risk at high doses is considered to be directly proportional to that at low doses. Direct and definite demonstration of thresholds is difficult. Once chemical hormesis is established, however, thresholds could be established unequivocally. In the current study, we demonstrate chemical hormesis. This study was conducted as a collaborative project of the Mammalian Mutagenesis Study Group (MMS) in the Japanese Environmental Mutagen Society (JEMS).

## Materials and methods

### Cells

Chinese hamster cells (CHL/IU) and human cells (HeLa S3 and TK6) were cultured in a CO_2_ (5%) incubator at 37 °C. The culture medium for CHL/IU and HeLa S3 cells was Eagle’s minimum essential medium (MEM) or Dulbecco’s modified MEM supplemented with 10% bovine serum or fetal bovine serum with or without penicillin and streptomycin. TK6 cells were cultured with RPMI 1640 supplemented with 10% fetal bovine serum. In the cytotoxicity tests, cells were dispensed with a multititer pipet, cultured in 96-well plates, and optical density was measured with a scanner. As an exception, 48-well plates were used.

### Cell activity detection reagent

Unless otherwise specified, 10 μL of the Cell Counting Kit-8 (CCK-8) (DOJINDO LABORATORIES, Kumamoto, Japan) were added to a 100 μL culture medium. CCK-8 contains 1-Methoxy PMS and WST-8. When 1-Methoxy PMS is reduced by cellular NADH and/or NADPH, this hydrogen carrier then changes colorless WST-8 to brownish colored WST-8 formazan, the absorption peak of which is 450 nm. Although named CCK-8 for ‘cell counting kit’, this mediates measurement not of cells but of cellular activity. Coloration was measured with a scanner at 450 nm. Unless otherwise specified, three wells per dose were used and the average was the basis of comparison.

### Mutagens

Mitomycin C (CAS: 1950–07-7) was purchased from Wako Pure Chemical Industries, Ltd. (Osaka, Japan). Ethyl methanesulfonate (CAS: 62–50-0) was from nacalai tecque, INC. (Kyoto, Japan).

### Rationale

**Fig. 1:**
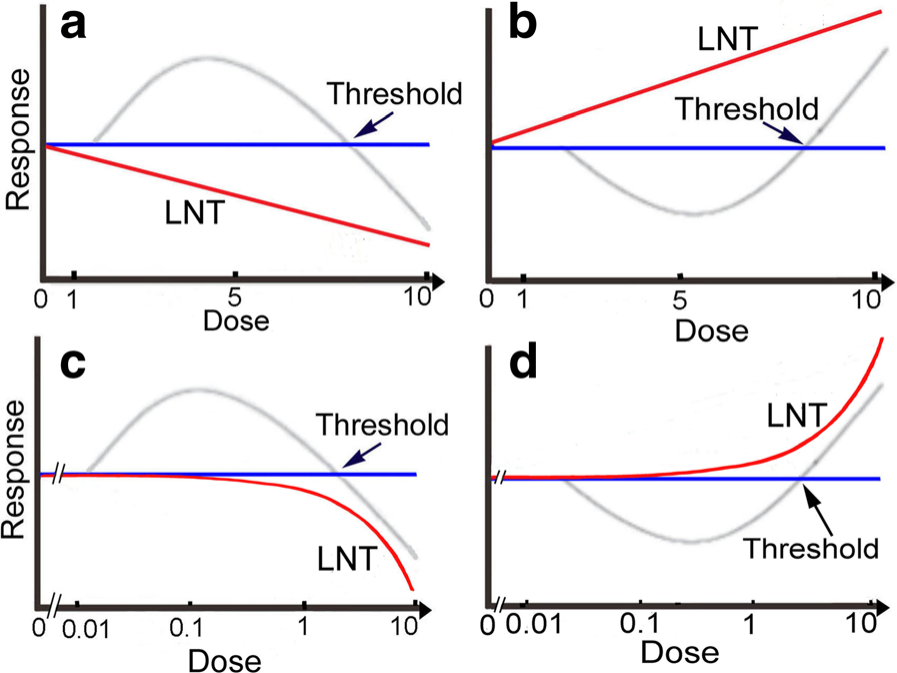
Schematic illustration of hormetic and LNT responses. Magnitudes of responses and doses are arbitrary. In hormesis, e.g., in cytotoxicity and cell killing tests, low doses are beneficial to cells and high doses are hazardous to them so that the dose-response curve shows a reverse U-shaped curve when plotted on a linear scale (**a**, **c**, gray line). Thus, a threshold could be determined at the cross-point of the curve and the x-axis. On the other hand, LNT assumes that toxicity increases dose-proportionally and therefore the dose-response relationship is depicted as a linear line (**a**, red line). When the x-axis is logarithmic, the linear line becomes a curved one (**c**, red line). It is quite important to note that all responses in LNT come under the zero dose level (**a**, **c**, blue line). In hormesis, e.g., in mutagenicity and carcinogenicity tests, responses are opposite to cytotoxicity and cell killing tests (**a**, **c**) and show a J-shaped curve (**b**, **d**, grey line). LNT responses follow a linear line upward from left to right when plotting on a linear scale (**b**, red line) or a curved line when plotted on a logarithmic scale (**d**, red line). Here again, LNT responses never come under the zero dose level (**b**, **d**, blue line)

Since direct demonstration of the presence or absence of thresholds is difficult, we made use of hormetic responses. The rationale is shown in Fig. 1. LNT assumes that responses plotted on a linear scale are linear to the zero dose (Fig. 1a, b, red line), while hormetic responses show a reverse U-shaped curve (Fig. 1a, c, gray line) or J-shaped curve (Fig. 1b, d, gray line) depending on detection systems. When a logarithmic scale is used, the linear line (Fig. 1a, b, red line) becomes a curved line (Fig. 1c, d, red line). When response curves are of reverse U- or J-shaped curves, we can establish thresholds at the cross-points of the curves and the x-axis.

## Results

### Preliminary tests

**Fig. 2:**
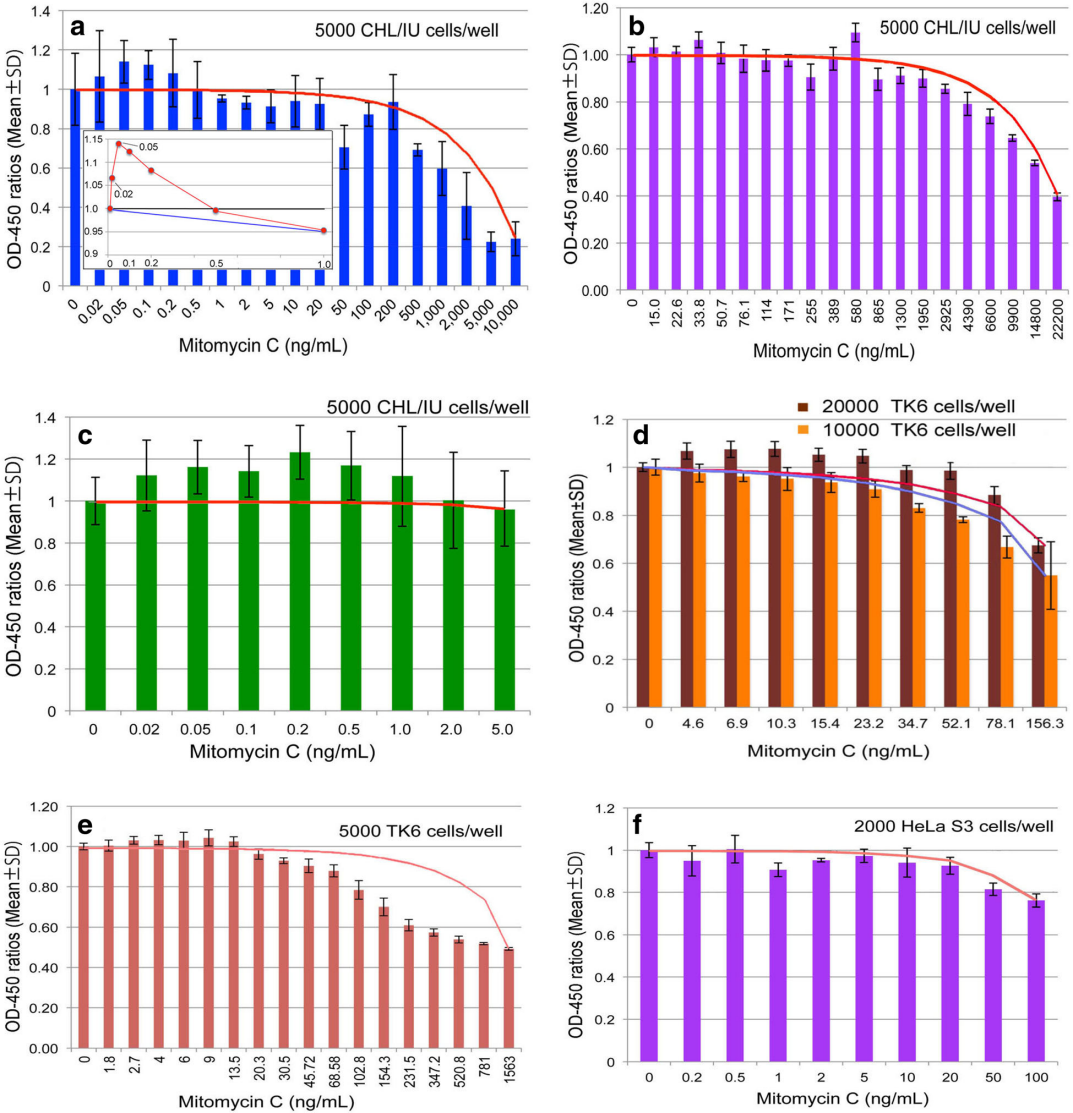
Preliminary experimental results. CHL/IU (**a**, **b**, and **c**), TK6 (**e** and **d**) or HeLa S3 (**f**) cells were treated with mitomycin C two days after inoculation for 24 h and OD-450 was measured 2 h after addition of CCK-8. Data show OD-450 ratios (Mean ± SD). Lines connecting between the zero dose and the highest dose depict dose-response relationship expected when applying the LNT hypothesis. Although the scale of the x-axis is neither linear nor logarithmic, it is closer to logarithmic; expectations from LNT are closer to curved lines as shown in Fig. 1c and d. When the scale of the x-axis is linear, presentation of all data in a single graph is difficult. Therefore, responses within a small range (from 0 to 1 ng/mL) are depicted on a linear scale in Fig. 2a (insert), in which the blue line shows an LNT response. The number of wells were three for **a** and **f**, four for **b**, **d**, and **e**, and six for **e**. In **e**, the outer 36 wells were used as medium blanks, and the mean reading was subtracted from experimental readings. The control in E consisted of 12 wells

At first, we attempted to find evidence of hormetic responses using three cell lines (HeLa S3, TK6, and CHL/IU), a test chemical (MMC), and a treatment time of 24 h. Results from the different participants are shown in Fig. 2. Firstly, one of the most important findings was that some lower doses induced higher cellular responses than those of the zero dose (Fig. 2a, b, c, d (20,000 cells/well), and E). Secondly, since higher doses were toxic to cells, a reverse U-shaped curve was seen (Fig. 2a, b, d (10,000 cells/well), and E), therefore indicating hormetic responses and plainly refuting LNT. Thirdly, when a wide range of doses were examined, dose response curves were not simple reverse U-shaped curves, but showed two or three peaks (Fig. 2a, b). This suggests that two to three cellular responses, such as repair and apoptotic mechanisms, are involved. When a narrower and smaller dose range was tested, dose response curves seemed to consist of a mild peak (Fig. 2 c and d (20,000 cells/well)).

Fig. 2 presents evidence rebutting LNT and allows setting of thresholds. There are some problems, however, in the hormetic responses, in that the responses were not always reproducible. For example, Fig. 2d shows that responses were hormetic in the 20,000 cells/well experiment, but not in the 10,000 cells/well experiment. Responses shown in Fig. 2f are not hormetic, though one- or two- peak responses can be seen. These non-hormetic responses were seen when the control values at the 0 dose level, as the basis of comparison, happen to be high. However, there must be some other confounding factors that cause fluctuations. Since subtle differences need to be detected in hormetic responses — stimulatory responses at lower doses and inhibitory responses at higher doses of the same chemical — possible confounders must be eliminated to the extent possible. Some possible factors are examined below.

### Air bubbles

The instruction of our scanner reads that air bubbles may disturb OD readings and should therefore be removed by brief centrifugation. Centrifugation, however, was not always useful to remove bubbles, especially small ones. If needed, bubbles were burst easily by putting a hot needle tip heated in the flame of a gas burner close to them.

### Edge effect

When 96-well plates are used, the edge effect — the possibility that water evaporates more from outer edge wells than inner ones, affecting cellular growth — has to be taken into consideration. When a preliminary test was conducted, the edge effect was seen 1 h after addition of CCK-8. The effect seemed to lessen after two hours; nonetheless, outer wells were not used in order to avoid this possible confounding factor. Since the outer 36 wells were discarded to avoid edge effects, 60 wells/plate were available. In testing, since many wells/dose and many doses/test are desirable, at least 3 wells/dose were used in this study.

### Convection effect

When cells in a well were examined under a microscope, the cells seemed to be denser in the area close to the wall than in the central area. This must be caused by convection. When suspended cells are inoculated and the plate is immediately put into a CO_2_ incubator at 37 °C, heat is conveyed mainly from the bottom rather than from the wall and convection occurs. Cells that floated up in the central area might sink down along the wall. If this occurs, cells in the central area become more sparse than in the peripheral area. When plates are left to stand for 30 min and cells are deposited on the substratum, convection of the medium would not disturb cellular distribution. Experimental results are shown in Table 1. Allowing the plates to stand for 30 min seemed to be effective for obtaining the even distribution of cells. This was confirmed by microscopic examination.

**Table 1:** Convection effect. CHL/IU cells were inoculated in wells of a 96-well plate (5000 cells/100 μL) or of a 48-well plate (10,000 cells/200 μL). After a two-day culturing period, 10 μL (96-well plate) or 20 μL (48-well plate) of CCK-8 were added to each well and OD-450 was measured 2 h after the addition of CCK-8

Plates	Standing time (min)	OD-450 (mean ± SD, *n* = 6)
96-well	8	0.826 ± 0.116
	30	1.085 ± 0.143
48-well	0	0.668 ± 0.022
	30	0.742 ± 0.004

### Coagulation of cells

When cells were inoculated in 96-well plates, cells cultured in dishes were removed by trypsinization and suspended in a 15 mL capped tube. An aliquot was allocated for cell counting with a hemocytometer. Cell counting and calculation for dilution takes around 15 min, during which time, cells might coagulate. Therefore, the cell suspension was divided into two further aliquots. One aliquot was left to stand and the other was reciprocally shaken at 60 rpm with a shaker. CHL/IU cells (5000) were plated and OD-450 was measured 2 h after addition of CCK-8 one or two days later. Results are shown in Table 2. The OD-450 was higher when cells were shaken. When cells were examined under a microscope, shaken cells showed more even dispersal than those that were left to stand. Cells were shaken in all experiments thereafter.

**Table 2:** Effect of conditions during cell counting on cellular activity

Measurement tim	Cells left to stand (OD mean ± SD, *n* = 15)	Cells shaken (OD mean ± SD, n = 15)
24 h	0.605 ± 0.087	0.634 ± 0.027
48 h	1.159 ± 0.090	1.371 ± 0.078

### Cell number per well and expression time

**Fig. 3:**
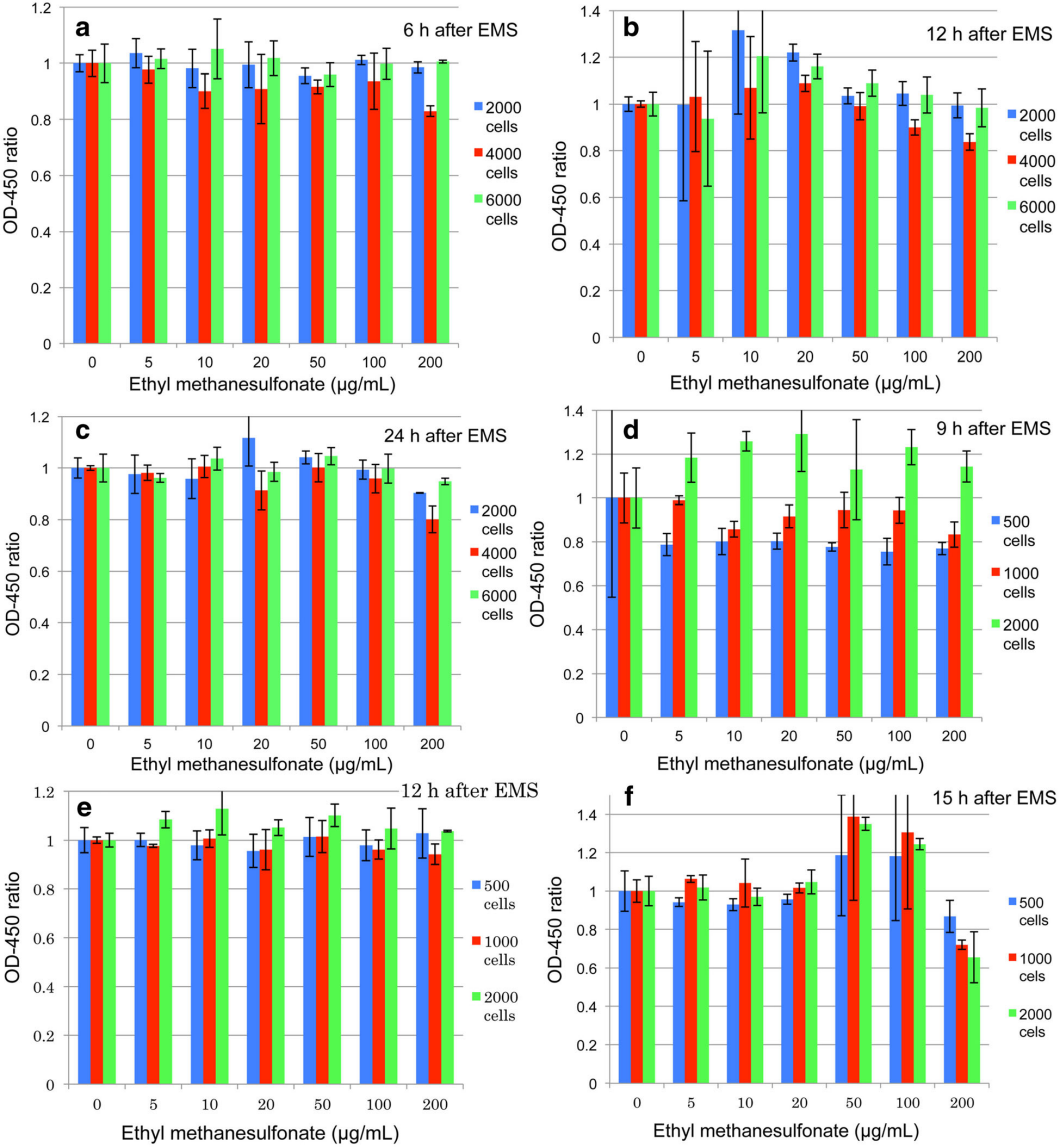
Number of cells per well and hormetic expression time. Firstly, different numbers of HeLa S3 cells (2000, 4000, and 6000 cells/well) were treated with EMS for 6 (**a**), 12 (**b**), and 24 h (**c**) two days after inoculation. Secondly, Hela S3 cells (500, 1000, and 2000 cells/well) were treated with EMS for 9 (**d**), 12 (**e**), and 15 h (**f**). CCK-8 (10 μL/well) was added 2 h before OD-450 measurement. Data show OD-450 ratios (Mean ± SD)

Hormetic responses could be seen 48 h after treatments, although responses tended to be lower as compared with 24 h treatments. Therefore, inoculum volumes and expression times were examined and the results are shown in Fig. 3. When 2000, 4000, and 6000 HeLa S3 cells were inoculated, responses were detected 6, 12, and 24 h after EMS treatments. Almost no responses were obtained after 6 h (Fig. 3a). An expression time of 12 h (Fig. 3b) showed higher responses than that of 24 h (Fig. 3c). As for cell numbers, 2000 cells per well generally responded higher than 4000 and 6000 cells. Next, 500, 1000, and 2000 cells per well were inoculated and responses were examined 9, 12, and 15 h after EMS treatments. The results showed that 2000 cells per well responded higher than 500 (Fig. 3d) and 1000 cells (Fig. 3e). From this, it was surmised that the number of cells must affect colorization of CCK-8. When the expression time was 15 h, (Fig. 3f), responses were almost equal between 1000 and 2000 cells/well. It is of interest to learn that hormetic responses were seen at higher doses after 15 h treatment (3f). This might show that biological responses differ depending on doses as hinted by the two - or three-peak responses shown in Fig. 2a, b.

A cell number of 2000/well was applicable to detect hormetic reactions in CHL/IU cells (fibroblasts), but not in TK6 cell (lymphoblastoids). More cells/well were appropriate for TK6; details will be reported elsewhere.

**Fig. 4:**
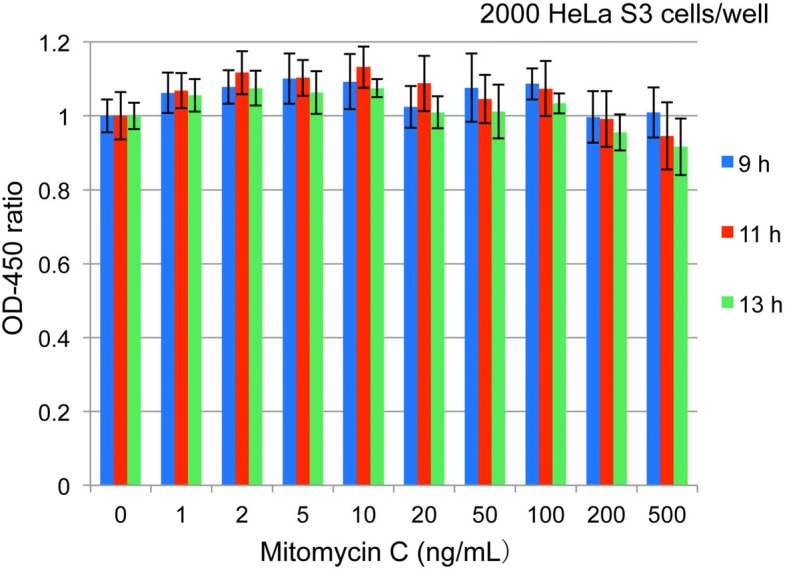
Re-examination of cell number and expression time. HeLa S3 cells (2000 cells per well) were plated and treated with MMC 48 h after plating. OD-450 was measured 9, 11, and 13 h after MMC treatment. Data show OD-450 ratios (Mean ± SD)

Fig. 3 suggests that the inoculum volume of 2000 per well and the expression time of around 12 h gives comparatively good responses. Therefore, 2000 cells per well were plated and OD-450 was measured 9, 11, and 13 h after MMC treatment. Results are shown in Fig. 4. There were no critical differences among the three detection times, and there seems to be two peaks.

### Nature of CCK-8

**Fig. 5:**
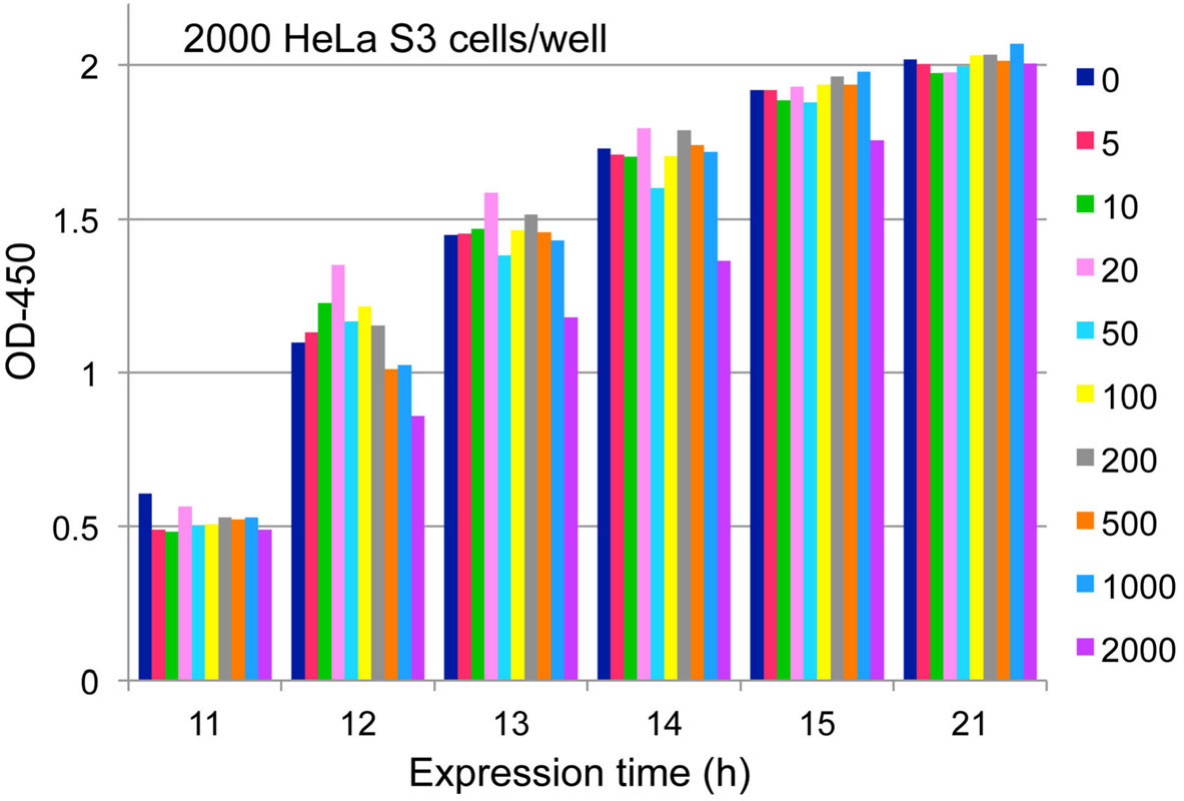
Characteristics of CCK-8 coloration. HeLa S3 cells (2000 cells per well) were plated and treated with nine concentrations (5 to 2000 ng/mL as shown at the right of the graph) two days after inoculation. CCK-8 (5 μL/well) was added 10 h after addition of MMC and OD-450 was measured 11, 12, 13, 14, 15 and 21 h after the addition of MMC (1, 2, 3, 4, 5, and 11 h after the addition of CCK-8, respectively)

Hormetic responses were measured using CCK-8 in this study. As indicated in its instructions, CCK-8 is stable for at least 24 h and is not cytotoxic. This was confirmed. Making use of these characteristics, the time course of OD-450 can be pursued over a long period. When 10 μL/well of CCK-8 was added to a well, OD-450 reached the saturation density in a couple of hours. Next, 5 μL/well were added and OD-450 was measured consecutively for several hours. Results are shown in Fig. 5. Coloration of CCK-8 was not sufficient at 1 h (11 h after treatment), with 2 h (12 h after treatment) seeming to be best for hormesis detection. OD-450 readings increased gradually and seemed to reach a plateau at 11 h (21 h after treatment), at which point hormetic responses disappeared. Thus, OD-450 measurement 2 h after addition of CCK-8 is recommended.

## Discussion

“Hormesis refers to adaptive responses of biological systems to moderate environmental or self-imposed challenges through which the system improves its functionality and/or tolerance to more severe challenges [27].” Various living organisms such as bacteria, alga, protozoa, plants, mice, and humans show hormesis [28]. In fact, all organisms would show hormesis if properly tested. The universality of hormesis indicates that this must be understood in the context of evolution. Life on Earth originated under radiation-rich and anaerobic conditions. The main action of ionizing radiation in living organisms is the production of reactive oxygen species (ROS) by ionizing water molecules. However, ROS are toxic to living organisms. Countering this, the process of photosynthesis started to produce oxygen around 2.5 billion years ago. Now, oxygen occupies 20% of the atmosphere. Oxygen is utilized to produce energy efficiently by aerobic metabolism, during the process which ROS — 10^9^/cell/day [29] — is produced. Without the detoxification mechanisms of ROS, aerobic organisms could not have evolved on Earth.

Respiration produces three orders of magnitude higher or more ROS than background radiation. Living organisms have evolutionally developed effective mechanisms to erase ROS. When the ROS barrier is broken and mutagenic lesions are formed in DNA, these are efficiently repaired by various repair mechanisms. When DNA lesions exceed the capacity of repair mechanisms, cells with these lesions are eliminated by apoptosis. When all these barriers are overcame and cancerous cells appear, the immune system eliminates not all but many of these cells. Neglect of these defense mechanisms is the pitfall in which lies the LNT trap. Just think if all radiation or carcinogens could induce mutations and if all mutations induced cancer.

Living organisms are surrounded by not only radiation and oxygen but also tens of thousands of organic and inorganic substances that may be toxic to them depending on dose levels. Living organisms have evolutionally developed defense mechanisms against these toxins, including mutagens and carcinogens. Since the LNT applied to mutagens and carcinogens is a failed hypothesis, we assume there must be thresholds in mutagenic action. Before testing mutagenicity, we examined thresholds in cytotoxicity tests. Thresholds could be established if hormetic responses take place, in which stimulatory activity at lower doses and inhibitory activity at higher doses appear (Fig. 1a, b). As stimulatory activity in hormesis is not very high and around 10% or so of the control, we tried to eliminate confounding factors to the extent possible. These included air bubbles, convection, the edge effect, and coagulation of cells. Medium changes or washing of cells is not recommended, because these processes were surmised to be confounders. As a result of this investigation, we could confirm hormetic responses in cytotoxicity tests with MMC and EMS and refute LNT at least in part.

It is of interest to learn that toxic effects at higher doses are more eminent than expected from LNT (Fig. 2a, b, and e). Apoptosis, a defense mechanism, may be involved in these responses in which severely damaged cells were eliminated.

Even if we took precautions in toxicity tests, data fluctuations make it difficult to attain reproducibility constantly. Since hormetic responses depend on the cells, test chemicals, dose levels, and other factors, some trial and error may be required to establish thresholds for a wide variety of chemicals. Still, we hope the present study is helpful as a starter protocol.

## Conclusions

The failed LNT is applied to mutagens and carcinogens. To provide evidence to rebut LNT, we tried to establish thresholds by utilizing hormetic responses in cytotoxicity tests using cultured mammalian cells. When MMC and EMS were used as test chemicals, thresholds were established. Based on these results, we put forward a tentative experimental protocol to detect chemical hormesis. This protocol is to inoculate 2000 cells at a volume of 100 μL per well, add various doses (10 μL, 3 wells/dose or more, especially for the control (0 dose)) of a test chemical 48 h after inoculation, add CCK-8 (10 μL) 10 h after treatment, and measure OD-450 2 h after addition of CCK-8. Since hormetic responses may depend on the nature of individual test chemicals and dose levels, some trial and error might be necessary to establish thresholds.

## Abbreviations

1-Methoxy PMS: 1-Methoxy-5-methylphenazinium methylsulfate
BEAR: Biological Effects of Atomic Radiation
BEIR: Biological Effects of Ionizing Radiation
CCK-8: Cell Counting Kit − 8
EMS: Ethyl methanesulfonate
EPA: U.S. Environmental Protection Agency
FDA: U.S. Food and Drug Administration
GP: Genetics Panel
JEMS: Japanese Environmental Mutagen Society
LNT: Linear no-threshold model
LSS: Life span study
MEM: Minimum essential medium
MMC: Mitomycin C
MMS: Mammalian Mutagenesis Study Group
NAS: U.S. National Academy of Sciences
OD: Optical density
RF: Rockefeller Foundation
ROS: Reactive oxygen species
SMH: Somatic mutation hypothesis
USA: United States of America
WST-8: 5-[2,4-Bis(sodiooxysulfonyl)phenyl]-3-(2-methoxy-4-nitrophenyl)-2-(4-nitrophenyl)-2H-tetrazole-3-ium
WWII: World War II
